# The Physician and Patient

**Published:** 1851-01

**Authors:** 


### ARTICLE I.

#### THE PHYSICIAN AND PATIENT.

This work which has received the commendation of most of our contemporaries in this country, and has been re-published in Europe, has just been laid upon our table by Mersrs Burley, Booksellers of Chicago. It is by Worthington Hooker, M. D., of Norwich, Connecticut, who seems to be conversant with "the mutual duties, relations, and interests of the Medical Profession, and the community," and has taken very just views of the errors of both parties, so far as a cursory glance through it will enable us to judge.

The work is written in an easy, fluent, and pleasing style, and does great credit to the taste and good sense of the author.

Those of our readers who have been accustomed to complain of the want in their knowledge and skill manifested by a portion of community, will here find the reasons for such incredibility; both those that unhappily arise from the uncertainty of Medicine, and those that depend upon deception and false appearances, which are fully, and we should think fairly discussed and philosophically explained. They will also find some excellent suggestions in reference to that line of conduct by which the physician should be guided, and by which he will attain to a position and standing that will enable him to become extensively useful.

The various forms of quackery are treated in a spirit of candor and with clear conceptions of their source, nature, tendencies, and a full and fair exposition of the pabulum of their support. A chapter too is devoted to the means of removing quackery.

The major part of the book which contains 552 pages, 12

mo., is devoted to the consideration of the influences and necessities of proper conduct on the part of the physician toward his patients, and of community towards him, to secure the most speedy and sure success in practice.

The closing chapter of the work is a consideration of "the trials and pleasures of a Medical life."

This chapter is full of truthful and pointed reflections, and of rich and pleasing consolations.

There is an appendix in the form of a letter on the subject of the propriety of the physicians paying attention to the spiritual wants of his patients, after which follows the code of etheics of the American Medical Association.

Altogether it is one of the most entertaining books that we have met with, and we cordially recommend our readers to procure it, and to try to get it into the hands of the intelligent members of their communities respectively, as it is calculated to diffuse correct information in a style that will be sufficiently attractive to secure a perusal. E.

